# Downstaging Conversion Therapy in Patients With Initially Unresectable Advanced Hepatocellular Carcinoma: An Overview

**DOI:** 10.3389/fonc.2021.772195

**Published:** 2021-11-18

**Authors:** Hui-Chuan Sun, Xiao-Dong Zhu

**Affiliations:** Department of Liver Surgery and Transplantation, Liver Cancer Institute and Zhongshan Hospital, Fudan University, Shanghai, China

**Keywords:** hepatocellular carcinoma, downstaging, conversion therapy, initial unresectable, systemic

## Abstract

The high mortality rate associated with hepatocellular carcinoma (HCC) is partly due to the high proportion of patients who present with advanced stage disease at diagnosis, for whom there are limited treatment options. For selected patients with initially unresectable HCC, locoregional and/or systemic treatments can result in tumor downstaging and consequently provide opportunities for surgical intervention and the potential for long-term survival. Therefore, the key aim of ‘conversion therapy’ is to reduce tumor burden so that patients become amenable to surgical resection. Various therapies have been investigated as candidates for downstaging patients with potentially resectable HCC including transarterial chemoembolization, transarterial radioembolization with yttrium-90 microspheres, radiotherapy, systemic therapies and combination or multimodality treatment approaches. However, downstaging conversion therapy remains controversial and there are several challenges such as defining the criteria used to identify the population of patients who are ‘potentially resectable’, the criteria used to define successful downstaging, and the optimum treatment approach to maximize the success of downstaging therapy. In this review article, we summarize clinical experience and evidence of downstaging conversion treatment in patients identified as having ‘potentially resectable’ HCC.

## Introduction

Worldwide, liver cancer is the sixth most commonly diagnosed cancer, with an estimated 905,677 new cases and 830,180 deaths in 2020 (1). The incidence rates associated with liver cancer are 2-3 times higher in men versus women (14.1 versus 5.2 per 100,000 individuals), with an overall mortality rate of 8.7 per 100,000 individuals (1). In China, Liver Cancer is the fourth most commonly diagnosed cancer, behind lung, stomach, and breast cancers, with 410,038 new cases in 2020 and 391,152 deaths (2). Similar to the global epidemiology, age-standardized incidence rates of liver cancer in China are higher in men than women (27.6 versus 9.0 per 100,000 individuals) and associated with an overall mortality rate of 17.2 per 100,000 individuals. Thus, both incidence and mortality rates for liver cancer are approximately 2-fold higher in China than global estimates (2). In addition, the estimated 5-year survival rate for Chinese patients with HCC is 12.2% (3).

The high mortality rate associated with HCC is partly due to the high proportion of patients who present with advanced disease, for whom there are limited treatment options (4). Surgical treatment provides the best opportunity for achieving long-term survival in HCC patients and is mainly comprised of hepatectomy and liver transplantation. For unresectable HCC, the application of preoperative treatments such as transarterial chemoembolization (TACE) may result in tumor downstaging and consequently provide initially ineligible patients with opportunities for surgical intervention. Improved long-term survival may be achieved in HCC patients undergoing resection after downstaging.

Downstaging conversion therapy is an emerging treatment approach for HCC that aims to reduce tumor burden using locoregional or systemic therapy so that patients become amenable to surgical resection. This type of preoperative therapy in HCC is controversial. However, evidence is accumulating to suggest that successful downstaging therapy followed by surgical resection is achievable in a subpopulation of patients. The criteria used to identify the population of patients who are ‘potentially resectable’, the criteria used to define successful downstaging, and the optimum treatment approach to maximize the success of downstaging therapy are all factors that remain subject to ongoing debate. This article will overview clinical experience to date with downstaging conversion treatment in patients identified as having ‘potentially resectable’ HCC. We also review clinical trials conducted in patients with unresectable HCC in which notable tumor responses were achieved, even if eligibility for resection was not reported as a treatment outcome.

## Conversion Therapy: Target Population and Principles

### Conversion Therapy – Target Patient Population

The causes of unresectable liver cancer can be divided into surgical causes and oncological causes. Surgical causes refer to the inability to perform safe surgical excision due to a patient’s inability to withstand surgery because of their general condition, liver function or insufficient remaining liver volume. Oncological causes refer to predicted efficacy after excision failing to surpass other, non-surgical treatment methods (5). There is a consensus for the definition of surgically unresectable liver cancer, while oncologically unresectable liver cancer is less well defined. The goal of conversion therapy is to eliminate these two causes, so as to achieve the conversion from unresectable liver cancer to resectable liver cancer (5).

**Figure 1:**
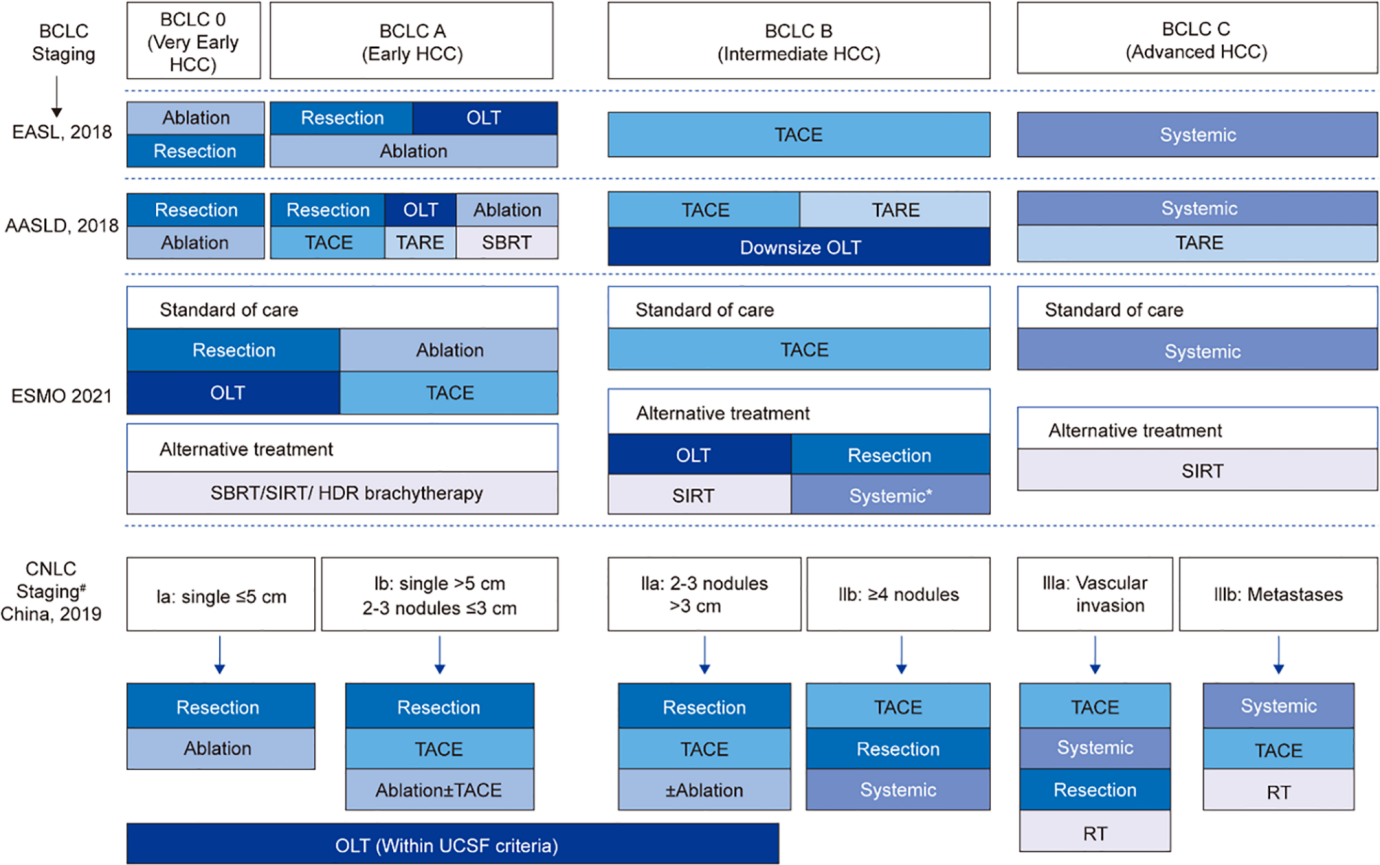
Comparisons of staging and treatment algorithms of HCC among 2021 ESMO, 2018 EASL, 2018 AASLD, and 2019 Chinese guidelines. *For patients who are not suitable for local therapies; ^#^If PS = 0-2, CNLC stage I-III; if PS > 2, CNLC stage IV; BCLC, Barcelona Clinic Liver Cancer; EASL, European Association for the Study of the Liver; AASLD, American Association for the Study of Liver Diseases; CNLC, China liver cancer staging; OLT, orthotopic liver transplantation; TACE, transarterial chemoembolization; TARE, transarterial radioembolization; SBRT, stereotactic body radiotherapy; SIRT, selective internal radiotherapy; RT, radiation therapy; UCSF, University of California San Francisco.

While the Barcelona Cancer Liver Clinic (BCLC) staging system for liver cancer is employed extensively throughout the US and Europe, in China the China Liver Cancer Staging (CNLC) system is preferred because of its relevance to local systems and practices (6). In general terms, the CNLC system divides each stage from the BCLC into two substages, with BCLC stages 0/A, B and C translating to CNLC stages Ia, Ib, IIa, IIb, IIIa and IIIb. BCLC stage 0/A is considered very early or early HCC and the equivalent CNLC stages Ia and Ib are defined as single nodules ≤5 cm or >5 cm, respectively; stage Ib also includes the presence of 2-3 nodules ≤3 cm. The intermediate BCLC B stage is represented within the CNLC system as stages IIa (2-3 nodules >3 cm) and IIb (≥4 nodules). The BCLC stage C, defined as advanced disease, is represented in the CNLC system by stages IIIa (vascular invasion) and IIIb (extrahepatic metastases) (6–8) (**Figure 1**).

Surgically unresectable CNLC-stage Ia, Ib, IIa liver cancer (considered unresectable mainly due to the patient’s general condition or liver function intolerance, insufficient remaining liver volume or insufficient resection margins) and surgically resectable CNLC-stage IIb and IIIa liver cancer (with limited tumor burden) are potentially resectable liver cancers, and multi-mode, high-intensity treatment strategies can be explored and adopted to facilitate the conversion. For surgically unresectable CNLC- stage IIb and IIIa liver cancer (for which the predicted surgical efficacy does not surpass other non-surgical treatment options), it is recommended that the current treatment norms should be followed and a gradual treatment strategy should be adopted, both the intensity and safety of treatment should be taken into account, and surgical excision should be conducted when applicable.

Conversion therapy may be considered distinct from neoadjuvant therapy. While both treatment approaches are administered as peri-surgical procedures and utilize the same modalities, they have different objectives. Neoadjuvant therapy is administered to patients with resectable disease to decrease tumor size prior to definitive surgery. In contrast, conversion therapy is administered to patients with initially unresectable disease who are considered potentially resectable after successful downstaging. However, when the treatment is applied to the patients with surgically resectable but oncologically unresectable HCC, both treatments may be overlapped in the target population (eg. surgically resectable CNLC-IIb or IIIa) or treatment objectives, eg. to change some oncological factors, such as tumor thrombi, satellite nodules, or even microvascular invasion. Basically, neo-adjuvant therapy is to decrease tumor burden or other oncological factors to improve the outcome after surgical resection, so neo-adjuvant therapy can be considered as oncological conversion therapy.

### Principles of Conversion Therapy

Conversion treatment strategies should be developed under the guidance of a multidisciplinary team, including surgeons, medical oncologists, interventional radiologists and diagnostic radiologists, or other related doctors. Conversion therapy planning should take multiple factors into account including liver function, liver function reserve, the number, location and size of liver lesions, vascular invasion, comorbidities, and the specific objectives of treatment. An ideal conversion treatment should have a high objective response rate and less adverse effects on patients and following surgical operation, and strive to achieve conversion in as short a timeframe as possible. During conversion therapy, the response to treatment should be closely monitored, and the timing of surgery should be determined based on a judgment of predicted efficacy, although an objective evaluation is needed to facilitate a good judgment.

Conversion therapy for downstaging or downsizing HCC can utilize many of the treatment modalities. Various therapies that have been studied as candidates for conversion therapy include TACE, transarterial radioembolization (TARE) with yttrium-90 microspheres (Y90), systemic therapies, and combination or multimodality treatment approaches (**Table 1**). An early literature review suggests that 8-18% of patients presenting with unresectable HCC may be suitable for salvage surgical resection after initial palliative treatment to downstage the tumor (23).

**Table 1:** Summary of clinical evidence for tumor down-staging in patients with unresectable HCC.

Ref	Study design	Treatment(patient No.)	ORR (%)	Downstaging rate or subsequent surgical rate (%)	Outcome
**Zhang et al., 2016 (** 9 **)**	Retrospective; single center; consecutive patients	TACE (831)	–	Downstaging rate: 9.9%	2-year OS rate: 93%
**Fan et al., 1998 (** 10 **)**	Retrospective	TACE (65)	–	100	5-year OS rate: 56%
**Labgaa et al., 2019 (** 11 **)**	Retrospective	TARE (349)	–	Subsequent OLT/LR rate: 9%	5-year OS rate:86%
**Tabone et al., 2020 (** 12 **)**	Retrospective; single center; consecutive patients	TARE (24)	–	20.8%	–
**Inarrairaegui et al., 2012 (** 13 **)**	Retrospective	TARE (21)	–	28.6%	–
**Lewandowski et al., 2009 (** 14 **)**	Single-center; comparative study	TACE vs TARE (78)	71% vs 86%	31% in the TACE group and 58% in the TARE group	–
**Zeng et al., 2002 (** 15 **)**	Retrospective	Radiation; hepatic artery ligation plus RIT vs TACE plus EBRT (67)	72% vs 86%	53% vs 23%	–
**Lee et al., 2014 (** 16 **)**	Retrospective consecutive patients	Concurrent chemo/radiotherapy (264)	–	6.8%	Curative resection group: 49.6% at 5-year survival
**He et al., 2019 (** 17 **)**	Randomized, open-label	Sorafenib vs sorafenib plus HAIC (247)	–	12.8%vs 0.8%	13.7m vs 7.13m
**Chong et al., 2018 (** 18 **)**	Retrospective	Concurrent chemoradiotherapy (CCRT) followed by HAIC	–	26.5%	–
**Zhu et al., 2021 (** 19 **)**	Single center retrospective study	TKI+PD-1	–	15.9%	–
**Zhang et al., 2021 (** 20 **)**	Single center retrospective study	TKI+PD-1+TACE	96%	56%	–
**He et al., 2021 (** 21 **)**	Randomized, open-label	Lenvatinib+PD-1+HAIC (LeToHAIC) vs. Lenvatinib	LeToHAIC Group: 67.6% (mRECIST)	LeToHAIC group: 12.7%	NR
**Zhang et al., 2020 (** 22 **)**	Prospective real-world study	Lenvatinib + PD-1	45.5% (mRECIST)	Conversion rate: 42.4%, surgical rate: 30.3%	NR

cTACE, conventional transarterial chemoembolization; DEB, drug-eluting bead; EBRT, external beam radiotherapy; HAIC, hepatic arterial infusion of chemotherapy; HCC, hepatocellular carcinoma; OLT, orthotopic liver transplant; ORR, objective response rate; OS, overall survival; PFS, progression-free survival; RT, radiotherapy; RIT, radioimmunotherapy; TAE, transarterial embolization; TACE, transarterial chemoembolization; TARE, transarterial radioembolization. NR, not reached.

## Locoregional Treatment for Conversion Therapy

### Transarterial Chemoembolization

TACE is the practice of delivering a chemotherapy agent directly to a liver tumor through the hepatic blood supply, usually the hepatic artery (24). To reduce leakage of the chemotherapy into the systemic circulation, microspheres loaded with chemotherapy have been developed (drug-eluting bead-TACE [DEB-TACE]). DEB-TACE permits sustained elution of the chemotherapy at the site of the tumor coupled with reduced systemic concentrations, allowing use of relatively high dose levels and/or frequency (24).

Data from a retrospective analysis show that patients who achieve tumor downstaging following TACE and then undergo surgical resection achieve better outcomes than patients who achieve downstaging but do not undergo surgery (9). Of the 831 patients with unresectable HCC included in this analysis who received TACE as initial treatment, 82 achieved significant downstaging and became eligible for resection. Of the patients eligible for surgery, 43 received salvage resection and 39 declined surgery. The majority of patients had an ECOG performance score 0-1 (91% and 87%) and were Child-Pugh class A (95% and 92%) in the group that received salvage resection and declined surgery respectively. Median overall survival (OS) was higher for patients who received surgery compared with those who declined (49 months versus 31 months P=0.027). A significant survival benefit favoring surgery was also achieved in subgroups of patients with macroscopic vascular invasion and with a partial response to TACE (9).

Several other studies have also indicated that TACE may be effective for downstaging tumors. In a retrospective study of patients with initially unresectable HCC who underwent hepatic resection following TACE, complete pathological tumor necrosis was achieved in 11 of 65 patients (16.9%) (10). Sixty-one patients in this series underwent resection, and 1-, 3- and 5-year survival rates were 80.0%, 65.0% and 56.0%, respectively (10). In addition, Li and colleagues reported that preoperative TACE did not impact perioperative morbidity or mortality in a multicenter, propensity matching analysis of patients with advanced HCC, 90.7% of whom had Child-Pugh class A liver function (25). Conversely, this study reported that preoperative TACE was associated with improved OS and relapse-free survival after liver resection in patients with large HCC (≥10 cm) (25).

The prospective randomized PRECISION V study compared response rates in patients receiving transarterial doxorubicin delivered *via* conventional TACE versus DEB-TACE (26). The ECOG performance score ratio (0/1) and Child-Pugh classification ratio (A/B) were similar (80/28 Vs. 74/19 and 89/19 Vs. 77/16) between the groups receiving TACE versus DEB-TACE, respectively. At 6 months, DEB-TACE was associated with significantly higher rates of complete response compared with conventional TACE (27% versus 22%), contributing to a higher overall response rate (ORR) for patients receiving DEB-TACE (52% versus 43%). In addition, the improved response rates among patients receiving DEB-TACE were more pronounced in the subgroup of patients with advanced disease. This study did not assess post-treatment eligibility for resection and therefore the translation of response rates into resectability is unclear. While subsequent studies have assessed DEB-TACE as a bridging therapy prior to liver transplant (27–29), the role of this treatment modality as a conversion therapy prior to surgical resection requires further confirmation.

### Hepatic Artery Infusion Chemotherapy

Interestingly, interim results from a phase 3 study of neoadjuvant HAIC with FOLFOX in patients with resectable HCC (BCLC stage A or B) showed that HAIC monotherapy can reduce the incidence of microvascular tumor thrombi, and this may suggest a role for HAIC monotherapy as part of a conversion therapy strategy (30). A study of patients with HCC and portal vein invasion who received either sorafenib or sorafenib in combination with hepatic arterial infusion of chemotherapy (HAIC; oxaliplatin, fluorouracil and leucovorin) indicated that the combination modality may have potential as a downstaging approach for patients with potentially resectable disease (17). In this study, 16 of 125 patients receiving sorafenib plus HAIC subsequently underwent curative resection and three patients achieved a pathologic complete response. Furthermore, resection was also possible in one patient with initial disease progression on sorafenib alone, who then crossed over to the combination arm (17). Another retrospective study comparing lenvatinib monotherapy with lenvatinib combined with toripalimab and HAIC (LeToHAIC) for the treatment of advanced HCC, in patients with an ECOG score of 0 or 1, found that LeToHAIC combination therapy was associated with longer progression free survival, longer OS, higher ORR and more complete responses than lenvatinib monotherapy (21). In addition, 9 patients in the LeToHAIC group received curative surgical resection following tumor shrinkage. A further study by Zhang et al. of 34 patients with unresectable liver cancer who were treated with PD-1 inhibitors combined with TKI and TACE, reported a conversion resection rate of 56% (20).

### Transarterial Radioembolization With Yttrium-90 Microspheres

TARE with Y90 microspheres is a form of selective internal radiotherapy (31). Several studies have successfully demonstrated tumor downstaging with TARE in patients with unresectable HCC (11, 12, 32). In a review of 349 patients with unresectable HCC who received TARE, 48% of whom were Child-Pugh class C, 10 were subsequently able to undergo liver resection, and an additional 22 patients received a liver transplant (11). In this cohort, TARE was associated with a decrease in viable nodules and led to tumor downsizing and downstaging (based on BCLC staging criteria). A single-center study reported that around 20% of patients with unresectable HCC and portal vein thrombosis achieved downstaging following TARE and became eligible for surgery (12). Of the 24 patients included in this series, five received surgical resection following TARE, four underwent right trisectionectomy and one received a liver transplant. Median survival in the five patients was 54 months (95% confidence interval [CI]: 17-92) compared to median survivals of 30 months (95% CI: 18-42) and 11 months (95% CI: 8-14) in patients who achieved partial response/stable disease (n=8) and those with progressive disease (n=11) following TARE. In this series, high tumor absorbed radiation and low pre-treatment alpha-fetoprotein (AFP) levels were significantly associated with the probability of successful downstaging. A further similar analysis of 21 patients with United Network for Organ Sharing (UNOS) stage T3 HCC indicated that 6/21 patients were downstaged and eligible for radical treatment with curative intent following TACE (13). Of these six patients, three underwent resection, two received a liver transplant, and one received ablation and then underwent resection. In this series, patients who were treated radically were significantly younger and had higher tumor volumes than those who did not achieve radical treatment. Finally, a number of studies that assessed the role of selective internal radiation therapy (SIRT) as conversion therapy for unresectable liver cancer have been summarized in a literature review by Cucchetti and colleagues (33). These authors suggest that SIRT can lead to considerable downsizing of tumors, and also promote hypertrophy of the contralateral lobe. A complete response rate of approximately 10% in patients with HCC receiving SIRT and an objective response rate of ~40% were reported, coupled with a maximum contralateral hypertrophy above 40% (33).

There is evidence to suggest that TARE may offer advantages over TACE in terms of improved response rates and safety as a candidate modality for conversion therapy. In a non-randomized trial, TARE was associated with a higher response rate than TACE (partial response rates of 61% and 37%) and resulted in more patients being downstaged from UNOS T3 to UNOS T2 and becoming eligible for transplant (14). Almost all patients in this study had a Child-Pugh class of A (56% and 53%) or B (44% and 42%) across the TARE and TACE groups respectively. A retrospective analysis of patients treated with TACE or TARE over a 9-year period also indicated that TARE offered several advantages, including an improved response rate, longer time to progression, and less toxicity compared with TACE (18). In both the TARE and TACE groups most patients had a Child-Pugh class of A (54% and 55%) or B (44% and 43%). Zori and colleagues reported that TARE is associated with improved survival and less microvascular invasion, and required fewer administrations over the same time period compared with TACE (34). Finally, another advantage of TARE may be its ability to cause hypertrophy of the contralateral future liver remnant, which may be useful in potential candidates for resection with a small liver remnant (35–37).

### Radiotherapy

The potential benefit of adding external beam radiotherapy (EBRT) to TACE as a strategy for tumor downstaging has been evaluated in multiple studies. A retrospective analysis of 203 patients with unresectable HCC without tumor thrombus, lymph node involvement, or extrahepatic metastases indicated a significant improvement in objective response rate among patients who received TACE plus EBRT compared with those who received TACE alone, and a numerical increase in the number of patients who became eligible for resection (38). Patients were selected for the combination therapy according to physician preference, and in most cases (49/54) this was due to defective lipiodol uptake during the TACE procedure, assessed by follow-up computed tomography (CT) scan. Approximately 85% of the patients enrolled in this study had tumors measuring >5 cm and were considered unsuitable for curative treatment. Objective response rates were 76% (41/54) among patients receiving EBRT plus TACE and 30.9% (46/149) among those receiving TACE alone (P<0.001), and sequential resection rates were 20.4% (11/54) compared with 12.8% (19/149), respectively (P=0.177) (38). Another retrospective study from the same group compared hepatic artery ligation plus radioimmunotherapy (RIT with 131I Hepama-1) with TACE plus EBRT in patients with unresectable HCC (15). Within the RIT group 4 patients (11%) had portal vein thrombi compared to 7 patients (20%) in the EBRT group. Objective responses were achieved in 85% (30/35) of those in the TACE plus EBRT group, and sequential resection rates following treatment were 23% (8/35) in this group (15). In addition, a randomized clinical trial evaluated the efficacy and safety of TACE plus external beam radiotherapy (TACE-RT group) compared with sorafenib for patients with hepatocellular carcinoma and macroscopic vascular invasion (39). The TACE-RT group showed a significantly higher radiologic response rate than the sorafenib group at 24 weeks (15 [33.3%] vs 1 [2.2%]; P < .001), and curative surgical resection was conducted for 5 patients (11.1%) in the TACE-RT group owing to downstaging. Finally, one study evaluated the oncological outcomes and prognostic factors of surgical resection after downstaging with localized concurrent chemoradiotherapy (CCRT) followed by hepatic arterial infusion chemotherapy (HAIC) in HCC patients with portal vein tumor thrombosis (PVTT) (40). Among 98 patients in the CCRT group, 26 patients (26.5%) underwent subsequent curative resection. During the same study period, 18 patients with PVTT underwent surgical resection as the first treatment. Clinicopathological characteristics and oncological outcomes between groups were compared. The median follow-up period was 13 months (range 1-131 months). Disease-specific survival was significantly different between the resection after localized CCRT group and the resection-first group (40). Overall, these studies indicate that combination regimen based on radiotherapy may represent a useful adjunctive treatment for patients with potentially resectable HCC who are candidates for tumor downstaging.

## Systemic Therapy

Systemic therapies including tyrosine kinase inhibitors (TKIs), immunotherapy, and chemotherapy, are widely used in the palliative treatment of HCC. Traditionally, systemic therapies for HCC have been associated with relatively low response rates and therefore neoadjuvant and downstaging therapy have not been part of standard management protocols. However, recent advances in systemic therapy have seen improved response rates, leading to a re-evaluation of the value of systemic therapy in the conversion therapy setting (**Table 2**).

**Table 2:** Summary of efficacy data of systematic treatments for advanced HCC.

Line	Treatment regimen		Study name	n	OS (months)	PFS (months)	ORR^a^ (%)	TTR (months)	Grade ≥3 TRAE (%)
1L	Mono	Lenvatinib (Global) (41)	REFLECT	478	13.6	7.3	18.3	–	57.0
		Lenvatinib (China) (42)	REFLECT	144	15.0	9.2	21.5^b^	–	44.0
		Sorafenib (43)	ORIENTAL	150	6.5	–	3.3	–	47.7^d^
		Sorafenib (44)	SHARP	299	10.7	–	2.0	–	52.0^f^
		FOLFOX4 (45)	EACH	184	6.4	2.94	8.15	–	55.7^c^
		Donafenib (46)	ZGDH3	328	12.0	3.7	4.6	–	37.5
	Combo	Lenvatinib+nivolumab (47)	Study117	30	–	7.39^b^	54.2	–	60.0^c^
		Lenvatinib+pembrolizumab (48)	Keynote524	100	22.0	8.6	36.0	2.8	67.0
		Apatinib+camrelizumab (49)	RESCUE	70	20.3	5.7	34.3	1.9	77.4
		Bevacizumab+atezolizumab (50, 51)	IMbrave150	336	19.2	6.9	30.0	–	43.0
		Regorafenib+pembrolizumab (52)	–	22	–	–	29.0	–	86.0^c^
		Anlotinib+penpulimab (53)	–	31	–	–	31.0	–	19.4
		Sintilimab+IBI305 (54)	ORIENT 32	380	–	4.6	21.0	–	56.0^c^
		Lenvatinib+AK104 (55)	–	18	–	–	44.4	–	26.7
		Lenvatinib+CS1003 (56)	–	20	–	8.4	40.0	–	35.0^c^
		Avelumab+axitinib (57)	VEGF Liver 100	22	14.1	5.5	13.6	–	72.7
2L	Mono	Pembrolizumab (58)	KEYNOTE240	278	13.9	3.0	18.3	–	18.6
		Regorafenib (59)	RESORCE	379	10.6	3.4	6.5	–	50.0
		Cabozantinib (60)	CELESTIAL	470	10.2	5.2	3.8	–	67.7^e^
		Camrelizumab (61)	–	217	13.8	2.1	14.7	2.0	22
		Apatinib (62)	AHELP	261	8.7	4.5	10.7	–	77.4
		Tislelizumab (63)	RATIONALE208	249	13.2	2.7	13.3	–	14.5
	Combo	Cabozantinib+nivolumab+ipilimumab (64)	–	35	NR	6.8	29.0	–	71.0
		Cabozantinib+nivolumab (64)	–	36	21.5	5.40	19.0	–	47.0
		Nivolumab+ipilimumab (65)	–	50	22.8	–	32.0	–	53.0
		Durvalumab+tremelimumab (66)	Study 22	74	18.7	2.17	24.0	–	35.1

^a^According to RECIST v1.1.

^b^According to mRECIST.

^c^TEAE.

^d^Treatment-emergent serious adverse events (SAE).

^e^AE.

^f^SAE.

Several studies have reported high ORRs with lenvatinib, either alone or in combination with other systemic treatments, in patients with initially unresectable HCC, making this a promising option for conversion/downstaging therapy. In the phase 3 REFLECT trial, lenvatinib was associated with a significantly higher ORR than sorafenib in patients with advanced unresectable HCC who had not received prior treatment for advanced disease and were Child-Pugh class A (41). In this non-inferiority study, the ORR was 40.6% in patients receiving lenvatinib versus 12.4% in those treated with sorafenib (odds ratio [OR], 5.01 [95% CI: 3.59-7.01]; P<0.0001) by modified RECIST (mRECIST), and the ORR by RECIST was 18.8% versus 6.5% (OR, 3.34 [95% CI: 2.17-5.14]; P<0.001). A network meta-analysis compare response rates, survival outcomes, and safety of first-line systemic therapies for advanced hepatocellular carcinoma, and the results showed that lenvatinib is associated with the best ORR of all systemic therapies included in the analysis (67). A further study from Japan included 107 consecutive patients who underwent lenvatinib treatment for advanced HCC, the majority of patients in this study had an ECOG score of 0 (87.9%) and were Child-Pugh class A (92.5%). Of the 107 patients included, 16 subsequently received surgical intervention, and R0 resection was achieved in nine (8.4%) patients. Survival analysis confirmed that successful conversion to R0 resection was associated with a longer time to treatment failure (68). Thus, data from the studies suggest that lenvatinib may have utility as a conversion therapy in patients who present with advanced, initially unresectable HCC.

Results from several small sample studies also support combination therapy with TKIs combined with immunotherapy in the conversion treatment setting in patients with unresectable HCC. For example, Zhu et al. reported 63 cases of patients with initially unresectable liver cancer treated with PD-1 inhibitors combined with a TKI, 60 of whom were classed as Child-Pugh A, and the conversion resection rate was 15.9% (19). A further study by Lu et al. of 33 patients with unresectable liver cancer with measurable PVTT (by mRECIST) and no extrahepatic metastasis who were treated with PD-1 inhibitors combined with Lenvatinib, reported a conversion rate of 42.4% (22). Huang reported that the response of intrahepatic tumor was less significant than that of tumor thrombi when treated with the combination of lenvatinib and PD-1 antibody (69), which suggested locoregional therapy can be used to improve the control of intrahepatic tumor when tumor thrombi is necrotic.

## Multimodal Treatment Approaches

Multimodality treatment approaches combine two or more treatment modalities with the aim of improving clinical outcomes beyond those achieved with either modality alone. Combining multiple therapies may provide cumulative benefits in term of efficacy beyond those offered by either modality alone, but may also be limited by non-overlapping toxicity profiles inherited from both modalities. Studies on multimodality treatment approaches in the setting of HCC conversion therapy with the intent of HCC downstaging are currently limited.

Concurrent chemoradiotherapy has been assessed for downstaging patients with unresectable HCC (16). In this study, 264 patients received radiotherapy (45.0 Gy with fractional dose of 1.8 Gy) and concurrent intra-arterial chemotherapy with 5-fluorouracil (500 mg/day), 15 of whom (83.3%) were classified as Child-Pugh class A. Eighteen of these patients (6.8%) subsequently underwent hepatic resection after achieving a response,. At the time of surgery, six patients had complete response, 11 had partial remission, and one had stable disease. In this study, cases were considered resectable when tumor-free margins and sufficient remnant volumes were obtained without extrahepatic metastasis. Median time from chemoradiotherapy to resection was 6.2 months (range 1-21 months), and median OS and disease-free survival were 61.8 months and 24.1 months, respectively. Three patients remained without evidence of disease recurrence for >5 years and all three had complete or partial remission with 90-100% necrosis post surgery (16).

A network meta-analysis of different embolization treatment strategies for unresectable HCC suggested that chemoembolization combined with external radiotherapy or local liver ablation could significantly improve tumor response rates compared with embolization alone (70). The OR (odds ratio) for achieving an objective response relative to control was 142 (95% CI: 55.9-395.4) with TACE plus ablation and 13.9 (95% CI: 6.9-31.3) with TACE alone. However, all treatments assessed in this analysis also increased the risk for serious adverse events relative to control, with the largest increases seen in patients receiving two treatment modalities. For example, the OR for a serious adverse event relative to control in patients receiving TACE plus ablation was 11.7 (95% CI: 1.5-128.7), and 14.6 (95% CI: 4.7-67.7) in those receiving TACE alone (70).

## Discussion

Surgery remains the only potentially curative treatment modality for patients with resectable HCC and normal liver function; however, only a minority of patients with HCC are eligible for resection at diagnosis. Conversion therapy aimed at tumor downstaging can increase the proportion of patients with HCC who are eligible for surgical resection; however, this approach is not routinely recommended in clinical practice at present due to a lack of supporting evidence. Despite this, accumulating evidence suggests that selected patients may achieve adequate downstaging following TACE or TARE to enable surgical resection. Lenvatinib-based combination therapy is also a promising option for conversion therapy in patients with potentially resectable disease with encouraging ORRs reported in clinical trials enrolling patients with initially unresectable HCC. Multimodality therapy may increase the proportion of patients eligible to undergo surgery or other curative treatments and reduce disease recurrence rates, allowing patients to experience a cancer-free, and drug-free status with long survival and good quality of life (71). It should be mentioned that there are other non-tumor focused approaches to conversion therapy such as techniques aiming to increase residual liver volume by inducing liver hypertrophy such as portal vein ligation (PVS) (72–74), artery ligation (75), and associating liver partition and portal vein ligation for staged hepatectomy (ALPPS) (76, 77). However, limitations of these approaches include a relatively long time for liver hyperplasia following PVE, which increases the chance of further disease progression, and an increased risk of complications with the use of ALPPS (78).

Ultimately, data from large randomized controlled trials are still required to clarify the clinical benefit of conversion therapy in patients diagnosed with unresectable HCC and identify specific patient groups likely to benefit from this approach. There is also a need for clearer definitions regarding which patients should initiate downstaging protocols and what criteria should be met before resection can be attempted. This will become increasingly important if treatments aimed at downstaging begin to differ from those used for palliative care. For now, preliminary data support the concept of downstaging in patients with potentially resectable HCC, offering the potential to provide clinical benefit to a population of patients in urgent need of expanded treatment options.

## Author Contributions

H-CS and X-DZ contributed to the conception and conduct of this review article, drafted and revised the article. All authors contributed to the article and approved the submitted version.

## Conflict of Interest

H-CS received honorarium for consulting or lecture from Eisai, MSD, Bayer, Roche, Gilead, Abbott, Beigene, Hengrui, Innovent, TopAlliance.

The remaining author declares that the research was conducted in the absence of any commercial or financial relationships that could be construed as a potential conflict of interest.

## Publisher’s Note

All claims expressed in this article are solely those of the authors and do not necessarily represent those of their affiliated organizations, or those of the publisher, the editors and the reviewers. Any product that may be evaluated in this article, or claim that may be made by its manufacturer, is not guaranteed or endorsed by the publisher.
